# Maternal feeding practices predict weight gain and obesogenic eating behaviors in young children: a prospective study

**DOI:** 10.1186/1479-5868-10-24

**Published:** 2013-02-18

**Authors:** Rachel F Rodgers, Susan J Paxton, Robin Massey, Karen J Campbell, Eleanor H Wertheim, Helen Skouteris, Kay Gibbons

**Affiliations:** 1 Department of Counseling and Applied Educational Psychology, Northeastern University, Boston, MA, 02115, USA; 2 Laboratoire de Stress Traumatique (EA- 4560), Universite Paul Sabatier, Toulouse, France; 3 School of Psychological Science, La Trobe University, Melbourne, Victoria, 3086, Australia; 4 Centre for Physical Activity and Nutrition Research, Deakin University, Melbourne, Victoria, 3125, Australia; 5 School of Psychology, Deakin University, Melbourne, Victoria, 3125, Australia; 6 Nutrition and Food Services, Royal Children’s Hospital Melbourne, Melbourne, Victoria, 3052, Australia

**Keywords:** Obesity, Child, Feeding practices, Eating behaviors, Body mass index

## Abstract

**Background:**

Maternal feeding practices have been proposed to play an important role in early child weight gain and obesogenic eating behaviors. However, to date longitudinal investigations in young children exploring these relationships have been lacking. The aim of the present study was to explore prospective relationships between maternal feeding practices, child weight gain and obesogenic eating behaviors in 2-year-old children. The competing hypothesis that child eating behaviors predict changes in maternal feeding practices was also examined.

**Methods:**

A sample of 323 mother (mean age = 35 years, ± 0.37) and child dyads (mean age = 2.03 years, ± 0.37 at recruitment) were participants. Mothers completed a questionnaire assessing parental feeding practices and child eating behaviors at baseline and again one year later. Child BMI (predominantly objectively measured) was obtained at both time points.

**Results:**

Increases in child BMI *z*-scores over the follow-up period were predicted by maternal instrumental feeding practices. Furthermore, restriction, emotional feeding, encouragement to eat, weight-based restriction and fat restriction were associated prospectively with the development of obesogenic eating behaviors in children including emotional eating, tendency to overeat and food approach behaviors (such as enjoyment of food and good appetite). Maternal monitoring, however, predicted decreases in food approach eating behaviors. Partial support was also observed for child eating behaviors predicting maternal feeding practices.

**Conclusions:**

Maternal feeding practices play an important role in the development of weight gain and obesogenic eating behaviors in young children and are potential targets for effective prevention interventions aiming to decrease child obesity.

## Background

The dramatic rise in overweight and obesity in children in the Western world has led to its identification as a major public health concern [1]. Overweight and obesity in children is associated with adverse health outcomes [2,3], and persists into adulthood [4]. Specific early parental (especially maternal) feeding practices have been proposed as important contributors to the development of obesity and obesogenic eating patterns (e.g., overeating) in children [5]. However, to date little prospective data exist regarding these relationships. Furthermore, the majority of existing research has focused on children over 5 years old and few investigations exist in younger children at the age when eating behaviors develop [6,7].

Parental feeding practices aim to influence the amount or type of food a child eats and include monitoring intake, restrictive or controlled feeding, pressure to eat, and instrumental and emotional feeding. These practices, although well-intended, can be associated with both child weight and obesogenic eating behaviors [5] – that is eating behaviors associated with higher calorie intake and overweight, including eating in absence of hunger, lack of caloric compensation, and a fast eating rate [6]. As feeding practices are potentially modifiable risk factors, understanding relationships between parent feeding practices and child weight and eating behaviors is of importance. However, to date, there has been a lack of clearly defined constructs describing maternal feeding practices, as existing measures present considerable overlap. We aim to clarify a set of core independent constructs representing maternal feeding styles and to identify which components most influence child body mass index (BMI) and eating behaviour.

Previous studies focusing on relationships between maternal feeding practices and child outcomes have produced inconsistent findings and focused on disparate concepts. Maternal monitoring, that is keeping track of one’s child’s eating, could be expected to result in healthy weight and eating behaviors, particularly at a very young age when mothers still strongly direct their child’s eating. While self-reported parent monitoring has been shown in one study to prospectively predict lower BMI [7], more often studies have found no relationship with weight change [8-10].

Restrictive feeding practices involve regulating the type and amounts of foods eaten by children. Although parents may attempt to restrict their children’s eating to reduce weight and maintain health, theorists suggest restriction may lead to the opposite effect by encouraging children to seek out restricted foods and failing to help children regulate eating based on satiety [5]. Longitudinal studies have provided mixed results. Consistent with theorists’ expectations, maternal restrictive practices have predicted increased BMI *z*-scores from 5 to 7 years [7], and greater eating when not hungry [11]. However, consistent with likely parental intentions, maternal restriction has also predicted *decreased* BMI [10,12]. Finally, other authors have not found maternal restrictive feeding practices predicted changes in child adiposity [8,9]. One potential explanation for these disparate findings is that different scales have been used to operationalize restrictive feeding. To address this, the present study included five frequently used measures of maternal feeding practices to uncover separate dimensions of maternal feeding before exploring their relationships with child obesogenic eating behaviors and weight change in a sample of younger children.

Parental feeding practices aiming to control when, where and what children eat have also been examined. A distinction has been proposed between overt and covert methods of controlled feeding and their respective influence on child weight and eating behaviors [13]. Overt control refers to explicit control over food consumption, such as being firm about what a child should eat. In one of the few prospective studies of very young children, overt control predicted increased BMI in children aged 12 months [14]. Covert control refers to the extent to which parents manage children’s food environments and restrict access to unhealthy foods, such as avoiding keeping snack foods in the house. Maternal covert control has been cross-sectionally associated with unhealthy snacking in children [13,15]. However, longitudinal explorations of overt and covert controlled feeding practices in relation to child weight status or obesogenic eating behaviors are lacking.

Another feeding practice, pushing to eat, might be expected to predict weight gain over time, although this has not been observed consistently. Among children from birth to 2 years old, as well as in a sample of 5 year olds, maternal pressure to eat has been found to predict lower BMI one year later [7,10], increases in child BMI [16], or to reveal no association with weight change or eating outcomes [8,9]. Maternal pushing to eat has also been associated with greater fat intake, which in turn was associated with increased BMI [17]. Prompting and encouragement to eat more or try new foods has also been considered in relation to child weight status and obesogenic eating behaviors [18]. However, the little existing cross-sectional research has shown an inconsistent association between encouragement to eat and child BMI [19,20], and further research is warranted.

Feeding practices which promote the use of food for reward or emotional regulation have also been postulated to influence child weight status and obesogenic eating behaviors. One such practice is instrumental feeding which involves using food as a reward for a desired outcome (e.g., in return for good behaviour). It has been suggested that reinforcing positive associations of palatable foods through instrumental feeding could strengthen the preference for reward (usually high-calorie) foods [21]. In one cross-sectional study, maternal instrumental feeding was associated positively with children’s snacking behaviors [22]. Furthermore, associations between maternal instrumental feeding and food enjoyment and food responsiveness in 3 to 6 year-olds have been found [23] but no significant association was found between instrumental feeding and child BMI *z*-scores in the only cross-sectional study that examined this relationship [19]. Although data is lacking, emotional feeding, i.e., using food to help a child regulate emotions, has also been posited to be associated positively with child weight status and obesogenic eating behaviors as a result of encouraging children to eat in the absence of hunger [19,20,22].

In summary, the existing literature examining parental feeding practices as predictors of changes in child weight and obesogenic eating behaviors is inconsistent and lacks longitudinal studies in very young children. Measurement issues may partially account for these discrepancies. Furthermore, most studies have focused solely on BMI as an outcome, rather than considering child eating behaviors that potentially contribute to overweight.

The principle aim of the present study was therefore to explore the role of a wide range of maternal feeding practices in relation to weight gain and obesogenic eating behaviors in a sample of 2 year-old children. To date, a variety of potentially overlapping constructs exist in the literature. Consequently, a preliminary aim was to conduct a principal components analysis to clarify a set of core independent constructs representing maternal feeding styles. Next, we aimed to identify which components of mothers’ feeding practices prospectively predicted the development of obesogenic child eating behaviours and BMI. We hypothesized that restrictive and controlling maternal feeding practices would be associated positively with increases in child BMI *z-*score over time, and we expected these maternal feeding practices to be associated positively, both cross-sectionally and prospectively, with obesogenic child eating behaviors. In order to test the competing hypothesis by which child eating behaviors predict changes in maternal feeding practices, we also examined these relationships prospectively.

## Methods

### Participants

Participants were 323 parent–child dyads recruited in 2008 (Time 1). A subsample of the first 284 dyads recruited was invited to participate in the second wave of the study a year later (Time 2). Of these, 222 accepted (78% retention rate). Children were aged between 1.5 to 2.5 years (mean age = 2.03 years, ± 0.37) at recruitment. Mothers (mean age = 35 years, ± 0.46) were included if they were over the age of 18 years and had a child between 1.5 and 2.5 years old, could read and understand English, and had children with no food allergies, intolerances or deficiencies. Mothers, on average were well educated, and had a medium household income (Table 1). 

**Table 1:** Ranges, means and standard deviations for BMI and child eating behavior dimensions at time 1 and time 2

	**Range**	**Time 1 Mean (SD)**	**Time 2 Mean (SD)**
		**N = 323**	**N = 222**
**Education**			
University course completed		73.7%	65.4%
Some additional training		9.0%	10.0%
Secondary school completed		11.8%	11.9%
Some secondary school		5.0%	10.3%
**Income**			
Under $20,000		2.7%	0.8%
$20,000 to $60,000		22.5%	20.8%
$61,000 to $100,000		37.0%	37.0%
$101,000 to $140,000		18.0%	19.2%
Over $140,000		19.8%	20.8%
Child BMI z score		0.30	0.10*
Satiety responsiveness	4-20	3.07 (.57)	3.14 (.59)*
Slowness in eating	3-15	2.86 (.72)	2.95 (.67)**
Food fussiness	4-20	2.63 (.75)	2.77 (.79)***
Food responsiveness	8-40	2.20 (.65)	2.26 (.60)*
Enjoyment of food	5-25	3.77 (.75)	3.74 (.67)
Desire to drink	3-15	2.57 (.80)	3.06 (.70)***

*p < .05, **p < .01, ***p < .001.

### Measures

#### Anthropometric assessment

Body Mass Index (BMI). Child height (to the nearest .1 of a centimetre) and weight (to the nearest .1 of a kilogram) were obtained and children’s BMIs were calculated and converted into a standardized *z*-score [24] adjusting for age and gender (referred to as BMI*z*). Children were lightly clothed for all measurements with shoes removed. All children were weighed by the researcher at Time 1. At Time 2, 64.1% were weighed by researchers but due to resource limitations, 35.9% were not. In the latter cases, maternal health records were used if the child had been measured within the last 3 weeks or parent report was used as last resort.

#### Maternal feeding practices

Five main measures assessing maternal feeding behaviors were identified which contained subscales related to monitoring, restriction and control, pressure or prompting to eat, and instrumental and emotional feeding.

Child Feeding Questionnaire (CFQ [25]). Maternal restriction and monitoring of children’s food and energy intake were measured using the Restriction and Monitoring subscales from the Child Feeding Questionnaire [25], which has been demonstrated to have good validity, reliability [26] and internal consistency [9,27] in children aged 2 to 11 years. The eight item Restriction subscale assesses the degree to which parents attempt to restrict their child’s food intake and eating during meals. The three item Monitoring subscale assesses the degree to which parents keep track and notice their child’s food. Each item is rated on a 5-point Likert scale ranging from 1 (disagree) to 5 (agree).

Preschooler Feeding Questionnaire (PFQ [28]). The five item Pushing to Eat subscale was used to assess parents’ inclination to pressure their child to consume more food. The four item Using Food to Calm subscale was used to assess parents’ tendencies to provide children with food as a means of regulating their negative affect. Each item is rated on a 5-point Likert scale ranging from 0 (never) to 4 (always). This scale has been shown to have good internal validity in a preschool sample [29].

Parent Feeding Style Questionnaire (PFSQ [20]). The four item Instrumental feeding subscale was used to assess the degree to which parents used food as a reward. The five item Emotional feeding subscale assesses the degree to which parents used food to regulate their child’s negative affect. The ten item Control subscale was use to assess the degree to which parents control their child’s eating patterns. Each item was rated on a 5-point Likert scale ranging from 1 (never) to 5 (always). These scales have demonstrated good test-retest reliability and internal reliability in preschool samples [20].

Control Over Eating Questionnaire (COEQ [13]). Overt forms (firmness regarding child’s eating patterns) and covert forms (control of the food environment) of parent control over children’s eating were measured using the five item Overt Control and the five item Covert Control subscales of the COEQ. Each item is rated on a 5-point Likert scale ranging from 1 (never) to 5 (always). These scales have demonstrated good internal reliability in children aged 4 to 11 years [13].

Comprehensive Feeding Practices Questionnaire (CFPQ [29]). The seven item Restriction for Weight Control subscale was used to assess parents’ restriction of food, particularly high sugar and fat foods in order to control their child’s weight. The three item Food as a Reward subscale assesses parents’ use of food as a reward for good behaviour. The five item Control subscale assesses parents’ tendencies to control what and when their child ate. The four item Modelling subscale assesses parents’ efforts to model the eating of healthy foods. This scale has been shown to possess good psychometric properties in samples of children aged 2 to 8 years old [29].

#### Child eating behaviors

The Child Eating Behavior Questionnaire (CEBQ [30]) was used to assess food approach and avoidant eating styles in children. The scale has previously been shown to have good psychometric properties in children aged 3 to 8 [31-33]. The present study used 6 of the original 8 subscales: the five item Enjoyment of Food subscale, the five item Food Responsiveness subscale, the three item Desire to Drink, the nine item Satiety Responsiveness/Slowness in Eating subscale, and the four item Food Fussiness subscale. The other two subscales were not used due to significant overlap with other included measures. Each item was rated on a 5-point Likert scale ranging from 1 (never) to 5 (always). Items from each subscale were summed and averaged to create a final score.

The parent version of the Dutch Eating Behaviour Questionnaire (DEBQ-P [34]) was used to assess external and emotional eating styles in children. The emotional eating subscale (13 items) was used to assess children’s tendencies to eat in response to negative affect. The external eating subscale (8 items) was used to assess children’s eating in response to food-related stimuli, regardless feelings of hunger. The items are scored on a 5-point Likert-type scale ranging from 1 (never) to 5 (very often). The parent version of the DEBQ has been shown to have good validity [34].

### Procedure

La Trobe Human Ethics Committee approval was received. Most participants were recruited from local playgroups within the greater metropolitan area of Melbourne (Victoria, Australia) at which a researcher presented study information and recruited interested mothers. A minority of participants were recruited from maternal and child health centres, leaflets, advertisement in local papers and word of mouth. Following written consent, children’s height and weight were measured at an interview at participants’ homes. Parents completed identical questionnaires at baseline (Time 1) and approximately 52 weeks later (Time 2); questionnaires were returned by reply paid envelope. Mothers received a $10 gift card on each occasion.

### Statistical analyses

A square root transformation was conducted on Time 1 Weight-based restriction to improve normality. When transformations were insufficient, non-parametric statistics and robust methods were used. Data reduction was conducted using principal component analysis. A BMI*z* change score was computed by subtracting Time 2 BMI*z* scores from Time 1 values. Relationships between maternal feeding practices and BMI*z* change, as well as child eating behaviors were explored with correlational analyses at each time point. Prospective relationships between (1) maternal feeding practices at Time 1 and child eating behaviors at Time 2; and (2) between child eating behaviors at Time 1 and maternal feeding practices at Time 2, were investigated using hierarchical multiple regression analyses. Analyses exploring prospective relationships included only the subsample which completed both assessments (N = 222). One outlier was excluded.

## Results

### Participant characteristics

Mean self-reported maternal BMI was 24.21 (± 4.66) with 4.7% of mothers underweight, 60.3% of mothers in the healthy weight range, 24.4% overweight, and 10.6% obese (Table 1). At Time 1, 6.8% of children were underweight (BMI < 5^th^ percentile), 16.3% were overweight (85^th^ percentile ≤ BMI < 95^th^) and 11.1% were obese (BMI ≥ 95^th^ percentile). At Time 2, 7.2% of children were underweight, 15.3% were overweight and 4.5% were obese. Weight status of both mothers and children was comparable to national data, although at Time 1, our sample was slightly heavier than the national average [35,36]. There was no difference in BMI*z* at Time 1 between children who were included in the follow-up and those who were not. Descriptive statistics for eating behaviors and BMI*z* at Time 1 and Time 2 are shown in Table 1. BMI*z* decreased from Time 1 to Time 2, but scores on all eating subscales increased significantly. There was no difference in BMI*z* change score between children measured by researchers and those who were not (measured mean = −0.18 (SD = 1.16), not measured mean = −0.20 (SD = 1.14), *t*(218) = −0.12, *p* = .90).

### Analysis of scale components

To explore the components of maternal feeding practice and child eating behavior measures, two principal component analyses (PCA) were conducted. The first PCA was conducted on the 78 Time 1 maternal feeding practice items. Eigenvalues and the scree plot indicated a 9 factor solution explaining 48% of the total variance, with interpretable dimensions after rotation. The first factor, “Instrumental Feeding”, displayed high loadings of the 4 items of the PFSQ Instrumental Feeding subscale, the 3 items of the CFPQ Food as Reward subscale as well as 2 items from the CFQ Restriction subscale. On the second factor, “Encouragement”, the 8 PFSQ Encouragement/Prompting subscale items, 3 items of the CFPQ Encouragement to Variety subscale, and 3 items of the CFPQ Modelling subscale loaded. On the third factor, “Emotional Feeding”, the 5 PFSQ Emotional Feeding subscale items and 4 items of the PFQ Use of Food to Calm subscale loaded highly. The fourth factor, “Control”, revealed high loadings for the 10 items of the PFSQ Control subscale as well as 4 items of the COEQ Overt Control subscale. The fifth factor, “Covert Control”, displayed high loadings for the 5 CORQ Covert Control subscale items. On the sixth factor, “Monitoring”, the 3 CFQ Monitoring subscale items loaded. The seventh factor, “Pushing to Eat More”, revealed high loadings for the 5 PFQ Pushing to eat more subscale items. The eighth factor, “Fat Restriction”, revealed high loadings for 6 items of the CFQ Restriction subscale. Finally the ninth factor, “Weight-based Restriction”, displayed high factor loadings for the 7 CFPQ Restriction for Weight Control subscale items. The standardized regression scores resulting from these factors were used in subsequent analyses [37].

An identical PCA was conducted for the Time 2 maternal feeding items in an attempt to replicate and confirm the structure. For the Time 2 items, the eigenvalues and the scree plot indicated an 8 factor solution was the best fit, explaining 48% of the total variance, with interpretable dimensions after rotation. The main differences with the Time 1 factor solution was that the five items from the Preschooler Feeding Questionnaire (PFQ [28]) loaded onto the Instrumental Feeding factor, the 3 items of the Child Feeding Questionnaire (CFQ [25]) loaded on the Fat Restriction factor, while the five reversed items of the Parental Feeding Style Questionnaire (PFSQ [20]) separated to form a separate Control factor. The use of both regular and reverse scored items in questionnaires is intended to decrease response biases, however previous research has shown that such items may perform poorly in factor analysis [38]. For the following analyses we therefore excluded the factor composed of the reverse scored items, and included the remaining 7 factors. Again, the standardized regression scores resulting from these factors were used in subsequent analyses [37]. Using these scores there was no difference in maternal feeding practices between children who completed both time points and children who completed only Time 1 except on Fat-restriction (high in those who dropped out, *p* = .013).

A second PCA was conducted on the 47 child eating behavior items at Time 1 and Time 2 separately. At Time 1, a 3 factor structure was revealed that explained 44% of the variance, with easily interpretable rotated factors. The first factor, “Emotional Eating”, revealed strong factor loadings for the 13 items of the DEBQ-P Emotional Eating subscale. The second factor, “Food Approach”, revealed strong loadings for the 4 items of the CEBQ Enjoyment of Eating subscale, negative loading of the 9 CEBQ Satiety Responsiveness/Slowness of Eating subscale, and negative loadings of the 6 CEBQ Fussiness items. Finally the third factor, “Tendency to Overeat”, revealed strong loadings for the 8 DEBQ-P External Eating subscale items, and the 3 CEBQ Desire to Drink subscale items and the 5 items of the Food Responsiveness subscale. Repeating the analysis with Time 2 items revealed an almost identical factor structure with the exception of one CEBQ item which loaded moderately strongly on the Tendency to Overeat factor at Time 1 and weakly on the Emotional Eating factor but the reverse was true at Time 2. Again the standardized regression factor scores derived for Time 1 and Time 2 were used in the subsequent analyses. (The PCA summary is available from the authors on request).

### Cross-sectional and prospective relationships between maternal feeding practices and child eating behaviors and BMI*z* change

Findings from the correlation analysis (Table 2) revealed that, cross-sectionally at Time 1, child emotional eating was correlated positively with instrumental and emotional feeding practices, and correlated negatively with controlling feeding practices. Child Food Approach correlated positively with maternal Encouragement, Covert Control, and Weight-based Restriction, and negatively with maternal Instrumental Feeding, Fat Restriction and Pushing to Eat More. Furthermore, there was a tendency towards a correlation with Control. Child Tendency to Overeat was correlated with maternal Instrumental Feeding, Emotional Feeding, Covert Control, Fat Restriction and Weight-based Restriction. 

**Table 2:** Correlations between maternal feeding practices, BMIz change and eating behaviors (N = 222)

		**Time 1 child eating behaviors**			**Time 2 child eating behaviors**		
**Time 1 maternal feeding practices**	**BMI*z *change score**^**P**^	**Emotional eating**^**S**^	**Food approach**^**P**^	**Tendency to overeat**^**P**^	**Emotional eating**^**S**^	**Food approach**^**P**^	**Tendency to overeat**^**P**^
Instrumental feeding	.19**	. 16**	-.15*	.15*	.11	-.12^¶^	.10
Encouragement	.01	.01	.27***	.01	-.13^¶^	.24***	.15*
Emotional feeding	-.04	.39***	-.02	.15*	.35***	-.01	.19**
Control	-.08	-.15*	.11^¶^	.02	-.10	.08	.18*
Covert control	-.03	.02	.16**	.16**	-.04	.11	.04
Monitoring	.02	-.11^¶^	.04	.00	-.16*	-.15*	.02
Pushing to eat more	.00	.06	- .12*	.09	.03	-.07	-.05
Fat restriction	-.01	-.02	-.19**	.21***	.10	-.04	.21**
Weight restriction	-.08	.09	.18**	.13*	.12^¶^	.21**	.03

Note: ^P^ = Pearson product–moment correlation; ^S^ = Spearman rho correlation; *p < .05; **p < .01; ***p < .001; ^¶^ = *p* < .10.

Prospectively at Time 2, these relationships were somewhat weakened. Time 2 child Emotional Eating was correlated with Time 1 maternal Emotional Feeding and correlated negatively with Monitoring while Encouragement just failed to meet significance. Time 2 child Food Approach was correlated with Time 1 Encouragement and Weight-based Restriction and correlated negatively with Monitoring, while the correlation with Time 1 Instrumental Feeding just failed to meet significance. Time 2 Tendency to Overeat was correlated with Time 1 Encouragement to Eat, Emotional Feeding, Control and Fat Restriction.

In relation to our first hypothesis, child BMI*z* change was moderately and positively associated with Time 1 maternal Instrumental Feeding. However, this was the only significant relationship for BMI*z* change.

A second correlational analysis was conducted exploring the relationship between Time 1 child eating behaviors and Time 2 maternal feeding behaviors (Table 3). Prospectively, Time 1 child Approach was negatively correlated with Time 2 maternal Instrumental Feeding while Time 1 child Tendency to Overeat was positively correlated with Time 2 maternal Instrumental Feeding. Time 1 child Emotional Eating was positive correlated with Time 2 maternal Emotional Feeding. Time 1 child Food Approach was positively correlated with Time 2 maternal Encouragement. 

**Table 3:** Spearman correlations between child eating behaviors at time 1 and maternal feeding practices at time 2

	**Time 2 Maternal feeding practices**						
**Time 1 child eating behaviors**	**Instrumental feeding**	**Emotional feeding**	**Encouragement**	**Control**	**Covert control**	**Fat restriction**^**P**^	**Weight restriction**
Emotional Eating	. 09	.29***	-.03	-.01	.10	-.03	-.06
Food approach	-.33***	-.05	.22**	.03	.06	-.10	.13^¶^
Tendency to overeat	.30***	.05	.00	.01	.14^¶^	.08	.02

Note: *p < .05; **p < .001; ***p < .001; ^¶^ = *p* < .10.

Hierarchical multiple regression analyses were conducted for each Time 2 child eating behavior, with Time 1 scores for the relevant eating behavior entered in the first step to control for baseline scores, followed by the maternal feeding practices from Time 1 which displayed significant correlations with the Time 2 eating outcome in the second step so as to determine which predictor variables contributed unique variance (Table 4). Regarding child Food Approach behaviour, after controlling for Time 1 levels in step 1, in step 2 maternal Monitoring, Weight-based Restriction and Encouragement contributed significantly to the explained variance of Time 2 child Food Approach behaviour. However, maternal Monitoring was the only significant unique predictor (β = −.14, *p* = .005). Regarding the prediction of child Tendency to Overeat, after controlling for Time 1, maternal Emotional Feeding, Encouragement and Control feeding practices contributed significantly to Time 2 child Tendency to Overeat scores, with both Emotional Feeding (β = .14, *p* = .021) and Encouragement (β = .15, *p* = .012) emerging as significant unique predictors. Regarding child Emotional Eating at Time 2, after controlling for Time 1 levels, maternal Emotional Feeding and Monitoring significantly increased the explained variance in the second step but Emotional Feeding was the only significant unique predictor (β = .14, *p* = .012). No regression analysis was conducted for BMIz change as Instrumental Feeding would have been the only predictor. 

**Table 4:** Predictors of child eating behaviors at time 2

**Dependent variable (T2)**	**Predictors (T1)**	**Step**	**R**^**2**^	**R**^**2**^**Change**	**F**	**β**
Emotional eating	Emotional eating	1	.34	.34***	F (1, 203) = 109.01	.58***
	Emotional feeding	2	.36	.02*	F (3, 189) = 36.99	.14*
	Monitoring					-.01
Approach	Approach	1	.53	.53***	F (1, 191) = 214.00***	.73 ***
	Monitoring	2	.55	.02*	F (2, 191) = 58.81***	-.14**
	Weight restriction	2				.08
	Encouragement	2				.05
Tendency to overeat	Tendency to overeat	1	.27	.27***	F (1, 191) = 73.79***	.53***
	Emotional feeding	2	.32	.05***	F (4, 192) = 23.87***	.14*
	Encouragement	2				.15*
	Control	2				.10^¶^

*p < .05, **p < .01, ***p < .001, ^¶^ = *p* < .10.

As a final analysis, we conducted hierarchical multiple regression analyses, for each Time 2 maternal feeding practice, with Time 1 scores for the relevant feeding practice entered in the first step to control for baseline scores, followed by the child eating behaviors from Time 1. Findings revealed that, controlling for Time 1 maternal Instrumental Feeding, child Time 1 Approach (*β* = −.18, *p* = .003) was a significant negative predictor of Time 2 Instrumental Feeding and child Time 1 Tendency to Overeat was a significant positive predictor (*β* = .24, *p* = .001), *F* (4, 184) = 26.11, *p* < .001, *R*^*2*^ = .35. Furthermore, controlling for Time 1 maternal Emotional Feeding, child Time 1 Emotional Eating was a significant predictor of Time 2 maternal Emotional Feeding (*β* = .13, *p* = .047), *F* (4, 184) = 19.99, *p* < .001, *R*^*2*^ = .31. Finally, controlling for maternal Time 1 Covert Control, child Time 1 Emotional Eating was a significant predictor of maternal Time 2 Covert Control (*β* = .11, *p* = .039), *F* (4, 184) = 52.02, *p* < .001, *R*^*2*^ = .53.

## Discussion

Findings from this research support the importance of maternal feeding practices in relation to child weight gain and the development of obesogenic eating behaviors in young children. They provide evidence of the importance of considering maternal feeding practices in relation to obesity in childhood.

Our initial aim was to explore the factor structure of different measures of maternal feeding practices including instrumental feeding, encouragement, emotional feeding, control, covert control, monitoring, pushing to eat more, fat restriction, weight-based restriction. Our findings revealed that 9 dimensions best described these practices. Consistent with previous findings, overt and covert control described separate dimensions [17], suggesting that *direct control* of the amount and type of food a child eats is distinct from attempting to create a non-obesogenic food *environment*. Our findings also revealed a distinction between restriction of high-calorie foods (fat restriction), and restriction of children’s eating for weight control purposes (weight-based restriction). It could be argued that while the first might be directed towards health goals, the second is directed towards maintaining a low weight, perhaps for appearance considerations. A distinction between pushing to eat more and encouragement and reinforcement to eat more was also revealed. The failure to discriminate between these discrete dimensions may help explain previous discrepant findings regarding correlates of maternal restriction [10,11] and pressure to eat [10].

Restrictive feeding practices revealed complex relationships with child eating outcomes. Consistent with our hypothesis, mothers who reported restricting their child’s food for purposes of weight were more likely to report that their child tended to approach foods and to overeat at Time 1. Furthermore, restricting food for weight purposes was prospectively associated with food approach behaviors and revealed a similar trend with child emotional eating. In addition, mothers’ restriction of child fat intake and child tendency to overeat were correlated both cross-sectionally and prospectively. Similarly, mothers’ overt control was prospectively associated with children’s tendency to overeat at follow-up. These findings are consistent with previous work among older children [3,11], and provide support for the proposal that, although practices such as encouraging a child to eat less for weight control purposes, restricting snacks between meals and limiting access to high-fat foods, may be intended to prevent weight gain, they may be counterproductive and lead to obesogenic eating behaviors in children such as regularly overeating [6]. Interestingly, our findings also revealed negative cross-sectional relationships between fat restriction practices and child food approach. It may be that parents of children with lower levels of food approach see less need to restrict the consumption of high-fat foods.

This study also explored the prospective relationships between instrumental and emotional feeding and children’s weight gain and obesogenic eating behaviors. Findings revealed that maternal instrumental feeding was associated, cross-sectionally with child emotional eating, and tendency to overeat, as well as prospectively with greater child BMI*z* gains. Furthermore, controlling for Time 1 eating behaviors, maternal emotional feeding practices was a significant predictor of children’s emotional eating and tendency to overeat one year later. Maternal instrumental feeding may lead to increased preference and consumption of high-calorie snacks in response to external cues in children and thus to weight gain. Furthermore, emotional eating in children has been associated with overweight [39] and the onset of binge eating disorder in adolescence [40] and is therefore important in the context of child obesity. These findings suggest that using food as a reward or a means of decreasing levels of negative affect in children may be associated with long-term negative outcomes in terms of weight gain and dysfunctional eating patterns. Our findings are consistent with a causal relationship between feeding practices and change in BMI, rather than the opposite direction, in line with previous reports of the failure of BMI to prospectively predict maternal feeding practices among young children [41]. Furthermore, it may be that these relationships vary with age and that these patterns are not present in older children who live in a less controlled environment in which maternal feeding practices may be diluted, thus partly explaining previous failures to evidence these relationships.

Among our sample, maternal monitoring of children’s eating was prospectively negatively associated with both child food approach and tendency to overeat behaviors, and was a significant negative predictor of food approach when controlling for Time 1 food approach levels. Consistent with this finding, previous studies have described a negative relationship between monitoring and later BMI [3]. It has been suggested that monitoring occurs in response to concerns regarding children’s weight status [5]. Although this may be the case, our findings suggest that maternal monitoring of child high-calorie food intake may contribute to helping children develop healthy eating behaviors and regulate their desire for appetizing foods (i.e., food approach behaviors) and may convey to the child the idea that while such foods can be enjoyed they should not be consumed in large quantities or replace more healthy components of a daily diet.

Mothers’ encouragement feeding practices were associated both cross-sectionally and prospectively with their child’s food approach behaviors, and prospectively with children’s tendency to overeat. Mothers’ pushing to eat more, however, displayed only a weak negative cross-sectional association with food approach, and a negative trend with BMI*z* gain. These results are consistent with previous reports that parental pressure to eat was related to decreases in BMI [3,10], and suggest that pressure to eat may occur in response to concerns about a child being underweight. Encouragement and prompting to eat, however, may provide positive reinforcement when children eat well and contribute to tendencies to eat in response to external prompts rather than internal satiety signals.

We also examined the reciprocal relationship in which child eating behaviors predict maternal feeding practices. Our findings provide some partial support for this pathway. Child tendency to overeat predicted increases in maternal instrumental feeding, and child food approach tendencies predicted decreases in maternal instrumental feeding. Furthermore, child emotional eating predicted increases in maternal emotional feeding and maternal covert control. The findings regarding increases in covert control are interesting as this has been suggested to be a particularly helpful feeding practice for helping children to achieve a healthy diet [42]. These findings support previous accounts of a bidirectional relationship between maternal feeding practices and child eating behaviors [43] and suggest that this interaction should be taken into account both in future research and the development of interventions.

Limitations of this research include the relatively small sample and whilst efforts were made to recruit widely, our sample is not necessarily representative. In addition, use of maternal self-report measures for feeding styles, child eating behaviors and a minority of Time 2 height and weight, may have biased our data. Finally, the somewhat small amount of variance in child BMI*z* change and eating behaviors explained by parent feeding practices (2-5%) suggests that other factors not assessed in this study are also likely to predict these outcomes. It has been suggested that other maternal variables including general parenting, family functioning and parental comments [18,44,45] might influence child weight gain and eating behaviors.

## Conclusions

Our study explored prospective relationships between a wide range of maternal feeding practices and both child weight gain and obesogenic eating behaviors among children aged 2 to 4 years. Findings suggest that maternal feeding practices contribute to shaping weight and related eating behaviors early in life. In particular, instrumental and emotional feeding as well as restriction and encouragement to eat more may be related to weight gain and obesogenic eating behaviors in young children. Furthermore, maternal monitoring of children’s high-calorie food intake may contribute to shaping healthy eating patterns. While more longitudinal research would help confirm these mechanisms, the present findings suggest the usefulness of engaging parents of young children in obesity prevention interventions and helping parents implement feeding practices which shape healthy eating behaviors and effective strategies for weight control.

## Competing interests

No author has any competing interests to declare.

## Authors’ contributions

RFR identified the research question, analysed data and wrote the manuscript. SJP was lead PI on the supporting grant, played a major role in the design of the study, oversaw data collection and cleaning, and assisted in preparation of the manuscript. RM was project manager and co-ordinated data collection and entry. KJC, EHW, HS and KG were PIs on the supporting grant, were closely involved in the design of the study and instrument selection, and assisted in preparation of the manuscript and data interpretation. All authors read and approved the final manuscript.

## Authors’ information

RFR is a Postdoctoral Research Fellow at Northeastern University, Boston. SJP and EHW are Professors in the School of Psychological Science at La Trobe University, Melbourne. RM is a Research Officer in the School of Psychological Science at La Trobe University, Melbourne. KJC is Senior lecturer in Nutrition and Population Health, School of Exercise and Nutrition Science, Deakin University, Melbourne. HS is Associate professor in the School of Psychology, Deakin University, Melbourne. KG is Manager, Nutrition and Food Services, The Royal children’s Hospital, Melbourne.
